# Vascular Injury Accompanying Displaced Proximal Humeral Fractures: Two Cases and a Review of the Literature

**DOI:** 10.1155/2011/742870

**Published:** 2011-05-15

**Authors:** Martijn Hofman, Jochen Grommes, Gabriele A. Krombach, Bernhard Schmidt-Rohlfing

**Affiliations:** ^1^Department of Traumatology, University Hospital Aachen, Pauwelsstrasse 30, 52074 Aachen, Germany; ^2^Department of Vascular Surgery, University Hospital Aachen, Pauwelsstrasse 30, 52074 Aachen, Germany; ^3^Department of Radiology, University Hospital Aachen, Pauwelsstrasse 30, 52074 Aachen, Germany

## Abstract

We present two cases in which displaced proximal humeral fractures are accompanied by vascular injury. These Injuries are very rare but severe and the accompanying vascular impairment can have great clinical consequences. Therefore, we try to emphasize on the importance of thorough and accurate diagnostics, because it is obligatory for early diagnosis and improving the eventual outcome of these injuries. The specific order in treatment (internal fixation first or vascular repair first) depends on the severity of the accompanying vascular injury. The increasing use of endovascular procedures to treat vascular lesions is a very interesting development with several advantages, especially in elderly and multimorbid patients.

## 1. Introduction

Peripheral neurological injury in association with displaced fractures of the proximal humerus is not unusual. However, major vascular injury in association with these fractures is uncommon [1, 2]. Extremity fractures are accompanied by arterial lesions in 0.9%. Certain skeletal injuries, such as supracondylar humeral fractures, elbow joint dislocation fractures, supracondylar femoral fractures, and posterior knee joint dislocations, are far more often accompanied by vascular lesions, with a frequency of up to 10%. Finally, all fractures involving long bones are at higher risk for vascular injuries [3]. 

Because of the rarity of vascular lesions associated with proximal humeral fractures, accompanying arterial lesions can be easily overlooked, especially in fractures not readily associated with arterial injury. Nevertheless, the possibility of axillary arterial injury should be considered in every fracture of the proximal humerus with severe medial displacement of the shaft [1]. 

In this paper, we present two cases of closed proximal humeral fractures with accompanying vascular lesions due to fracture displacement and describe the treatment options. This paper was motivated in the first place by the rareness and severity of vascular complications accompanying a proximal humeral fracture. In second place by the fact that there is a considerable variation in clinical symptoms of these fractures. Furthermore, we wanted to show that endovascular procedures are valuable alternatives to open procedures with a minimum of additional trauma.

## 2. Case Report I

A 92-year-old woman was referred to the emergency room after she was found at home lying for approximately 2.5 hours at the floor after a fall on her left shoulder. The patient complained of pain in her left upper arm and of hypoesthesia of her left forearm distally to the elbow joint.

The initial clinical examination showed mild swelling of the proximal upper arm with tenderness on palpation or with attempted movement in the glenohumeral joint.

Vascular examination showed intact but weak brachial, radial, and ulnar pulses. The skin of the left hand and forearm was discoloured and cooler. The capillary refill was sluggish. The monophasic Doppler signals of both the ulnar and radial artery were weaker on the left side than on the right side. A reliable neurologic examination could not be performed because of the patient's pain. Deep tendon reflexes were normal on both sides. The clinical symptoms did not change after a single attempt of closed reduction.

**Figure 1:**
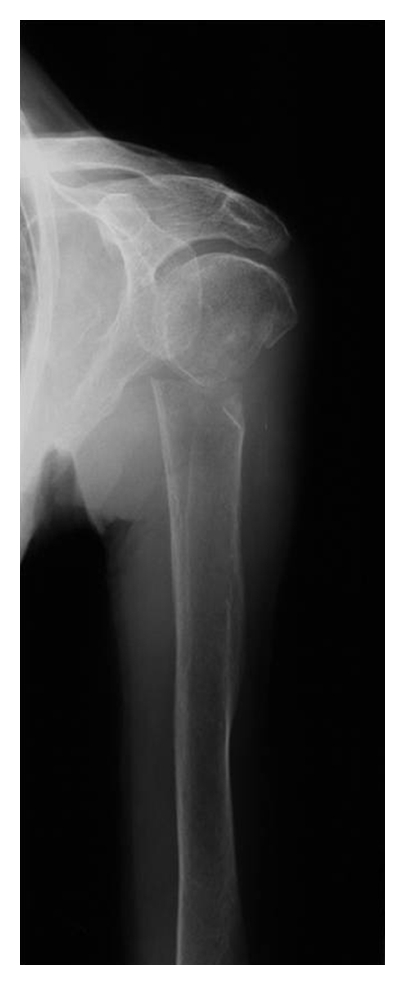
Initial X-rays of the dislocated proximal humeral fracture (AP-view).

**Figure 2:**
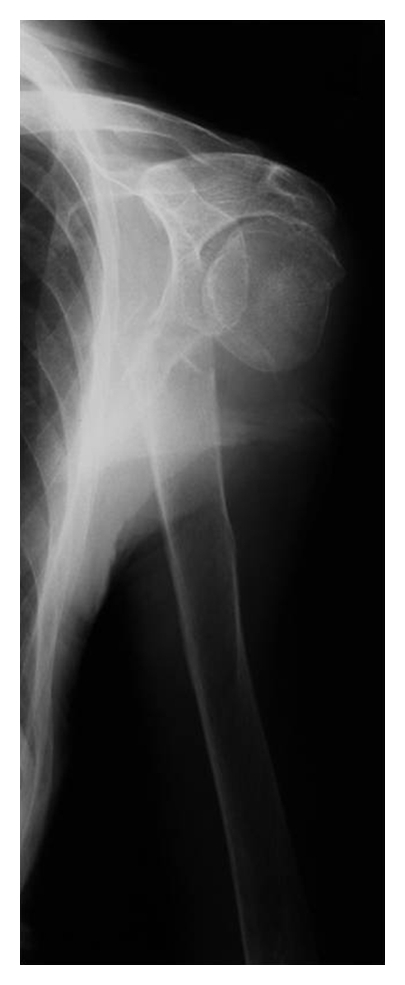
Initial X-rays of the dislocated proximal humeral fracture (Scapula-Y-view).

The initial radiologic examination showed a 2-part proximal humeral fracture with a complete displacement between the humeral head and shaft (Type 11-A3 according to AO-classification) (Figures 1 and 2).

**Figure 3:**
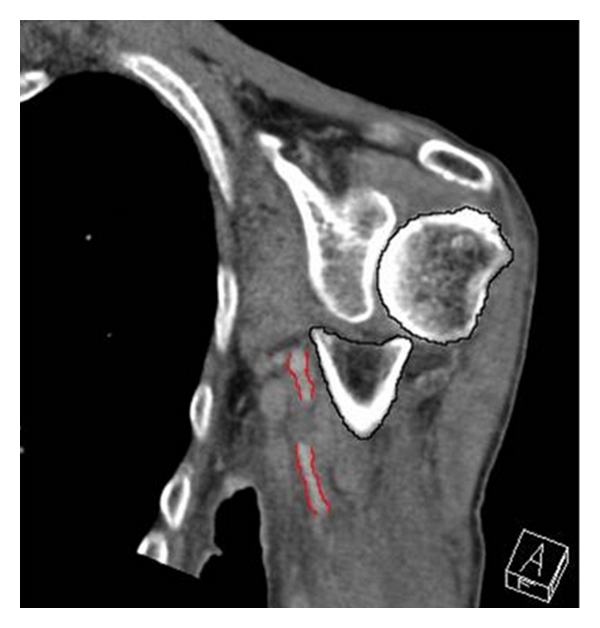
Interruption of the axillary artery (red) flow 2 cm caudal to the fracture (Humerus in black).

A CT-angiography was performed and demonstrated an interruption of the axillary artery flow just distal to the subscapular artery branch in the area of the branches of the Circumflex arteries with a surrounding hematoma corresponding to a possible intimal dissection of the axillary artery (Figure 3). 

**Figure 4:**
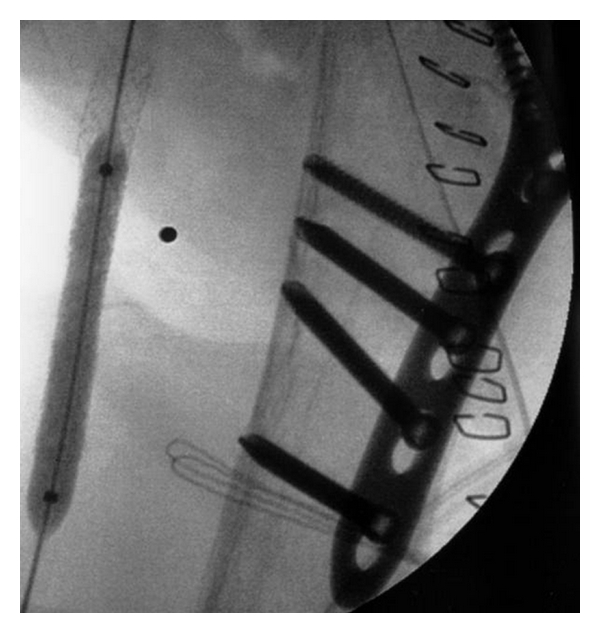
Implantation and dilatation of the Expert-stents in the axillary artery.

**Figure 5:**
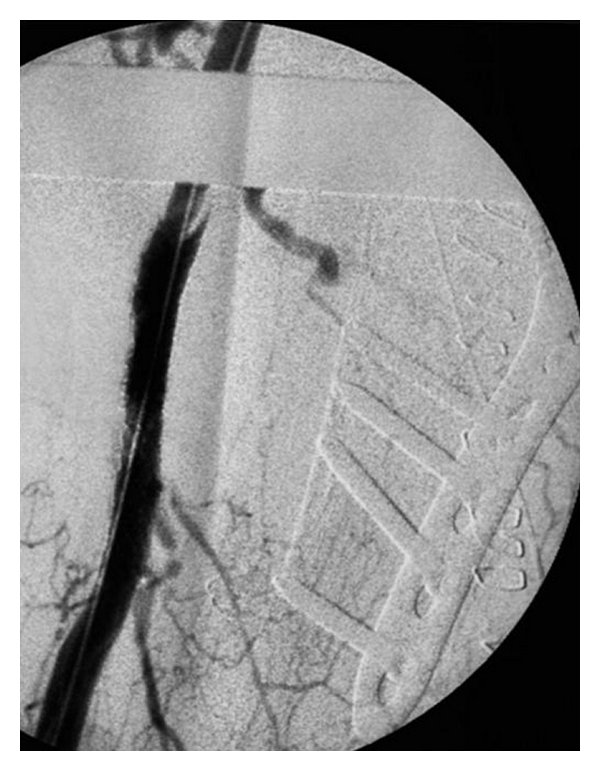
Restored vessel patency after stenting the axillary artery.

**Figure 6:**
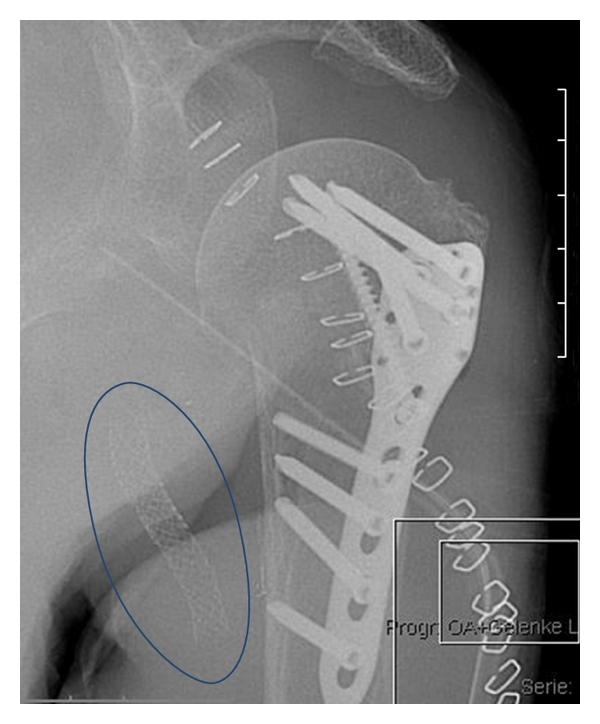
End result after Plate-osteosynthesis and stent implantation (blue oval).

After the CT-Angiography, the patient was operated on. First, an open reduction and internal fixation of the proximal humerus with a fixed angle plate osteosynthesis was performed through a deltopectoral approach. Subsequently, the vascular surgeons performed an arteriotomy and a retrograde thrombectomie of the axillary and brachial artery with a Fogarty catheter manoeuvre. A control-angiography was performed since the outflow was not restored and this confirmed a 5 cm long traumatic dissection of the axillary artery. Then two (6 × 30 mm) self-expanding stents were implanted (Figure 4). After this procedure, the vascularisation was restored and the brachial, radial and ulnar pulses fully returned (Figures 5 and 6).

Postoperatively, the patient complained of a transient motor deficit of the left hand muscles, which recovered a few days after surgery. One week postoperatively, the patient developed a pneumonia, which deteriorated the general condition to such an extent that she died a few days later.

## 3. Case Report II

**Figure 7:**
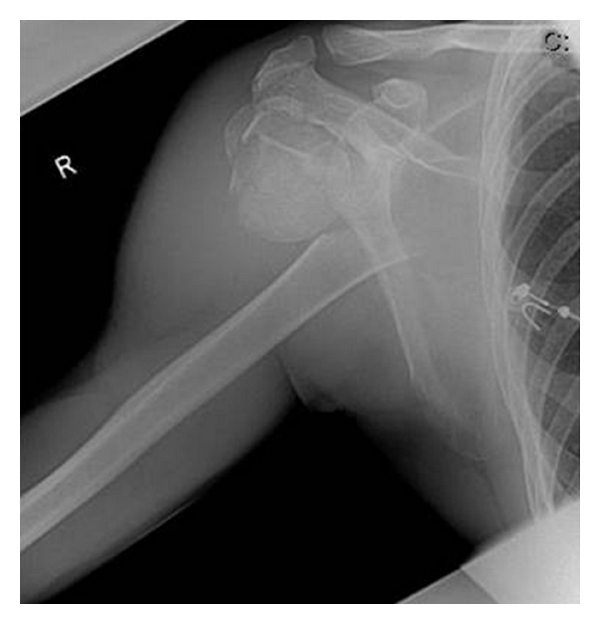
Initial X-ray of the dislocated proximal humeral fracture.

A 48-years-old male patient was transported to our hospital after a fall from 4 m height on his right shoulder. After the primary survey according to ATLS-standards, a fracture of the humeral head on the right side, type 11-B3 according to AO-classification, was diagnosed (Figure 7). The patient complained of paraesthesia of his right shoulder and digits 1–3 of his right hand. On palpation, both the radial and ulnar pulses were absent. A Doppler examination showed pulselessness of both the radial and ulnar arteries. After intubation and a single reduction attempt, the radial pulse was weak or absent, respectively, dependent on the position of the right arm.

Because of the dislocated fracture situation with compression of the brachial artery, an immediate operative therapy was indicated and performed.

**Figure 8:**
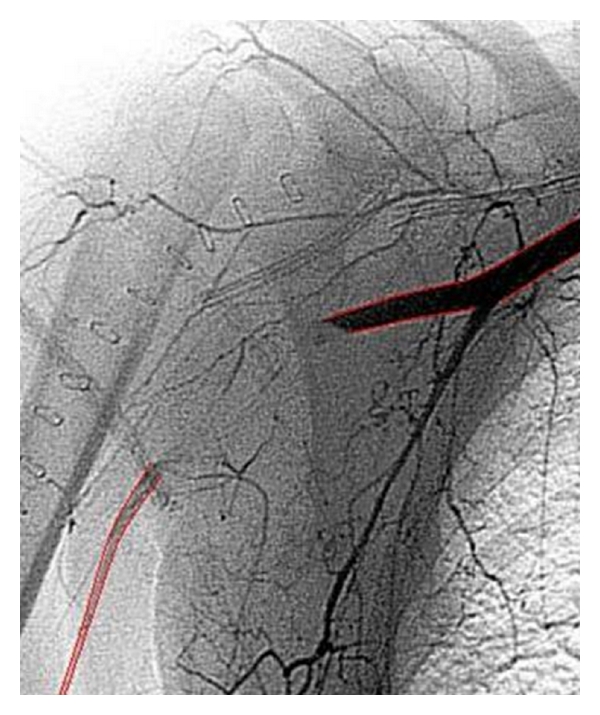
Collateral filling of the brachial artery.

An attempt of open reduction and internal fixation with a plate failed because of the comminuted fracture of the humeral head. Finally a humeral head resection was performed as preparation for an arthroplasty procedure. After the resection, we performed a digital subtraction angiography, which showed a 5.5 cm long dissection of the axillary artery. Through collateral vascularisation, the brachial artery faintly filled with a short delay (Figure 8). 

**Figure 9:**
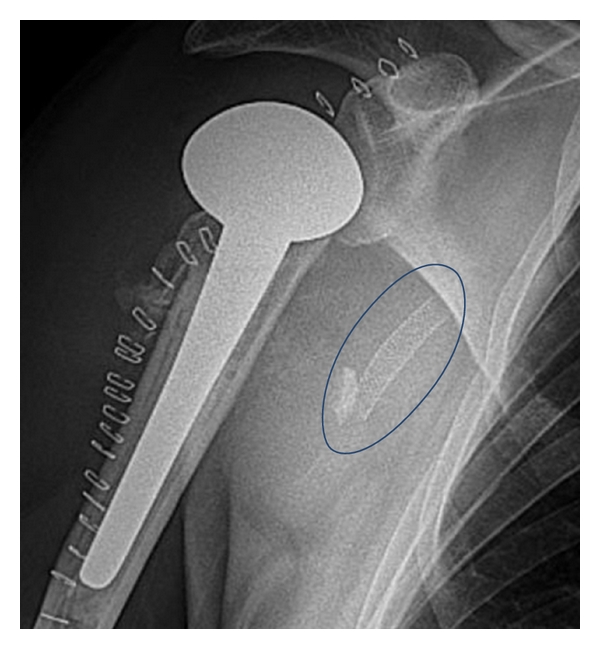
End result after prosthesis and stent implantation (blue oval).

**Figure 10:**
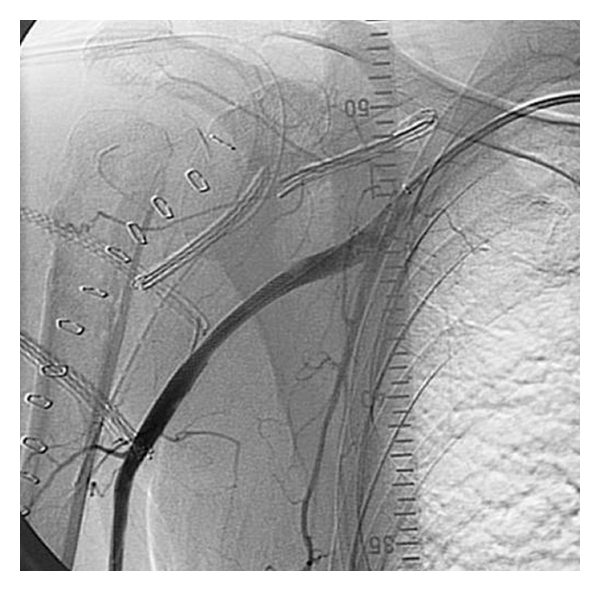
Restored vascularisation after stenting the axillary artery.

In the revascularisation procedure which followed, a 29 mm long self-expanding wall stent (Figure 9) with a diameter of 8 mm was positioned in the proximal part of the dissection. The control angiography showed a normal outflow of the axillary artery (Figure 10). After this endovascular intervention, a definitive shoulder hemiarthroplasty was performed. Postoperatively the patient was treated with an abduction orthosis and physiotherapy. The stent was protected with acetylsalicylic acid 1 × 100 mg and clopidogrel 1 × 75 mg.

## 4. Discussion

The primary cause of upper extremity vascular injury is a penetrating trauma, whereas only 5–10% of these injuries are caused by blunt trauma [4, 5]. Shoulder dislocation, hyperabduction, or clavicular fractures are the usual causes of blunt trauma to the axillary artery. Its association with closed proximal humeral fractures is rare [2, 4–7]. 

The most common vascular injuries are partial lacerations and complete transections. Partial laceration causes bleeding or formation of pseudoaneurysms. Complete transection leads to retraction and thrombosis of the ends of the vessels. Injuries to vascular structures can be caused by the following mechanisms: direct injury by fracture fragments, and injury by stretching or tenting of the artery over the fracture site [8, 9]. The subsequent ischemia is considered to be due to one or more of the following causes: spasm from kinking, thrombosis usually caused by intimal dissection, and overstretching of the artery [5, 6, 9]. Accompanying neurological deficits can be pathophysiologically explained by direct pressure on the nerve (bone fragment/hematoma) and/or compression or damage of the vasa nervorum, causing ischemic dysfunction [7]. The diagnosis of arterial injury accompanying a proximal humeral fracture is above all a clinical diagnosis, with a positive predictive value of 96% [6]. The clinical presentation depends on the type of arterial injury (Table 1). 

Clinical presentation can be occult because the axillary artery has 5 major branches providing excellent collaterals about the shoulder girdle. The clinical symptoms represent (at least partly) the classic six P's of Pratt (1954): Pulselessness, Paleness, Pain, Paresthesia, Paralysis, and Prostration. The most common physical finding is an absent or diminished pulse, but it is important to consider that a palpable pulse distal to the injury may be present for several hours after the injury, or never disappear at all due to the excellent collateral circulation around the shoulder [1, 6]. In a review, the extremity was pulseless in 75%, whereas pulses were decreased in 14%, and a normal pulse was palpated in 11% of the cases [6]. The aggregate of symptoms of an acute vascular occlusion depends on the extend of the occlusion, the extensiveness of collateral vessels, and the duration of the occlusion. After a critical period of 4–6 hours, irreversible damage can occur depending on the formation of collaterals.

If vascular compromise is suspected, a Doppler examination is required and if shock treatment and/or a single reduction of the fracture do not lead to restoration of the blood flow, an (CT-) angiography is indicated. Small arterial contusions with small limited intimal flaps may be diagnosed only by angiography. 

A major vascular lesion proximal to the elbow should be repaired (artery and/or vein) and the blood flow must be restored within 6 to 8 hours to salvage the compromised extremity [3, 8]. 

In case of a short ischemic time or a marginally compromised vascularisation, a rapid ORIF (open reduction and internal fixation) of the proximal humeral fracture allows direct vascular suture in a stable operating field and also prevents redisplacement and potential compromise of the vascular repair [1–3]. In case of a prolonged or critical ischemic period, the vascular repair must be performed at once and prior to the stabilisation of the fracture [10]. There are several treatment options to repair the vascular structures. Amongst thrombectomie and stenting, as shown in our cases, there are the following other options: conservative treatment in case of excellent collateralization, atherectomy with thrombectomy/endarterectomy, vascular reconstruction (suture, if the damaged segment is no longer than 1-2 cm), venous patch, partial resection with end-to end anastomosis, vein grafting, PTFE or Gore-tex grafting, and bypass of the axillary artery with a reversed saphenous vein graft [2, 4, 8, 10]. Ligation of the axillary artery should be prevented, because this results in a loss of the extremity in 42% of cases [9]. Although the optimal management of such injuries is unclear, the role of endovascular stent-grafting has expanded over the past decade. Endovascular treatment and stenting of arterial injuries with a bare or covered stent at the time of the operation or as a standalone procedure represents a minimal invasive approach. Due to the reduced soft tissue injury, the endovascular treatment has several advantages, including a faster recovery and less operative stress to elderly and multimorbid patients.

Principally, the end result can be further compromised by neurologic comorbidity and therefore, recognition of a possible neurologic impairment besides the associated vascular injury is vital to increase the functional outcome [10].

## 5. Conclusion

Displaced proximal humeral fractures with accompanying vascular and/or neurologic injury are rare but severe injuries with possible extensive clinical consequences. Even if the fracture and vascular reconstructions are performed with outstanding outcomes, the final result can be further compromised by neurologic damage. Therefore, in accordance to the literature, a thorough clinical examination of the neurovascular status is mandatory for early diagnosis and improving the eventual outcome of these injuries. Major fracture dislocations and high energy trauma should raise suspicion. The specific order in treatment (internal fixation first or vascular repair first) depends on the severity of the accompanying vascular injury. Endovascular treatment represents a minimal invasive approach and can be of strong advantage, especially in elderly and multimorbid patients.

##  Conflict of Interests

The authors declare that they have no conflict of interests, any grant, or financial profit related with this study.

## Figures and Tables

**Table 1:** Clinical presentation of arterial injury.

Type of injury	Clinical presentation
Partial laceration	Decreased pulse, hematoma, hemorrhage
Transection	Absent distal pulse, ischemia
Contusion	Initially normal, may progress to thrombosis
Pseudoaneurysm	Bruit or thrill decreased pulse
AV Fistula	
External compression	Decreased pulses
